# Navigating adoption barriers for microbial proteins in future food

**DOI:** 10.1038/s41467-026-73987-0

**Published:** 2026-06-10

**Authors:** Rima Gnaim, Hakimi Kassim, Lisa Neidhardt, Thomas Gassler, Rodrigo Ledesma-Amaro

**Affiliations:** 1 Imperial College Centre for Engineering Biology, Imperial College London, London, UK; 2 Department of Bioengineering, Imperial College London, London, UK; 3 Bezos Centre for Sustainable Protein, Imperial College London, London, UK; 4 Engineering Biology Mission Hub on Microbial Food, Imperial College London, London, UK; 5 Department of Biology, Faculty of Science and Mathematics, Universiti Pendidikan Sultan Idris, Tanjong Malim, Perak Malaysia

**Keywords:** Industrial microbiology, Metabolic engineering

## Abstract

Microbial biomass fermentation, in which microbes are cultivated to produce nutrient-dense biomass, offers a scalable route to sustainable protein production with low land, water, and greenhouse gas footprints. However, its shift from a speciality to a mainstream food continues to be difficult. Here, we move beyond technological overviews and propose a three-phase adoption framework: novelty barrier, early trust-building, and mainstream normalisation. This framework organises techno-economic, regulatory, and infrastructural barriers into a single trajectory. We trace single-cell proteins’ rise, decline and resurgence, map engineering and policy enablers by phase, and outline levers to move microbial proteins into resilient food systems.

## Abstract (web summary)

Microbial biomass fermentation offers a scalable route to sustainable protein production with a low environmental footprint. In this Perspective, the authors propose a three-phase adoption framework to help move microbial proteins into resilient food systems.

## Introduction

The global agri-food system is facing unprecedented strain from unhealthy diets, rising protein demand, and scrutiny of its ethical and ecological impacts^1^. Mainstream livestock-based protein sources are associated with substantial environmental burdens and face significant constraints in meeting rising demand without adverse consequences for human and environmental health^2^. Although environmental impacts differ markedly across agricultural systems, species, and production practices, the agri-food sector overall remains a major driver of land conversion, freshwater and marine eutrophication, and greenhouse gas (GHG) emissions.

Our food systems account for 26% of global CO₂-equivalent emissions and 78% of aquatic nutrient loading, and already occupy over half of the planet’s habitable land^3–5^. On an energy-input basis, animal-derived foods require 4–40 times more energy than the nutrition they deliver, a factor amplified by feed cultivation, nitrogen-rich manure production, antibiotic use, and pasture expansion through deforestation^6^. Livestock accounts for 68% of enteric N₂O, 64% of total NH₃, and 35-40% of total CH₄ emissions^7^. Despite these pressures, global meat consumption has increased by over 50% since the 1960s, reaching an average of 122 g person⁻¹ day⁻¹ in 2021^8^. The demand for animal proteins is projected to increase by approximately 110% between 2005 and 2050, reaching 465 million tons^9–11^. Animal-derived foods remain a major source of dietary protein worldwide, which helps explain both their continued importance in food systems and the urgency of developing more sustainable complementary and alternative protein sources^4^.

Alternative protein technologies, including plant-based analogues, cultivated meat, and microbial foods, have emerged as promising strategies to reduce reliance on animal agriculture and its associated environmental impacts. Here, microbial foods refer broadly to foods and food ingredients produced from microorganisms or through microbial processes for human consumption, including microbial biomass-derived products, fermented ingredients, and other microorganism-enabled food platforms^12,13^. For microbial proteins, cultivated fungi, bacteria, yeasts, and microalgae yield nutrient-rich biomass that typically contains around 30-70% protein on a dry-weight basis, together with dietary fibre. In mycoprotein, the fibre fraction is characterised by a chitin-glucan matrix, with glucan predominating and smaller amounts of chitin, alongside lipids, vitamins, and minerals^14–16^. Beyond its nutritional value, mycoprotein offers useful techno-functional properties, including a naturally fibrous structure, water-holding capacity, and structure-forming behaviour, allowing it to contribute to texturisation in food products and, in some applications, reduce reliance on conventional texture-building additives^16,17^. In addition, single-cell oils (SCO) enable the sustainable production of omega‑3 fatty acids, serving as alternatives to palm oil or cocoa butter for food and industrial uses^18^. Here, we use ‘microbial protein’ as the broader term for protein-rich foods, while ‘single-cell protein’ (SCP) is the traditional term used in earlier literature and historical contexts.

Despite the benefits of microbial foods and their technical feasibility, large-scale adoption remains a major barrier. This Perspective proposes a three-phase framework that enables the adoption of microbial proteins in future foods, integrating consumer psychology with techno-economic and policy constraints from novelty through to mainstream normalisation. This framework organises the microbial-protein transition into discrete psychological and policy phases, each tied to concrete engineering and governance levers (Boxes 1–3).

**Fig. 1:**
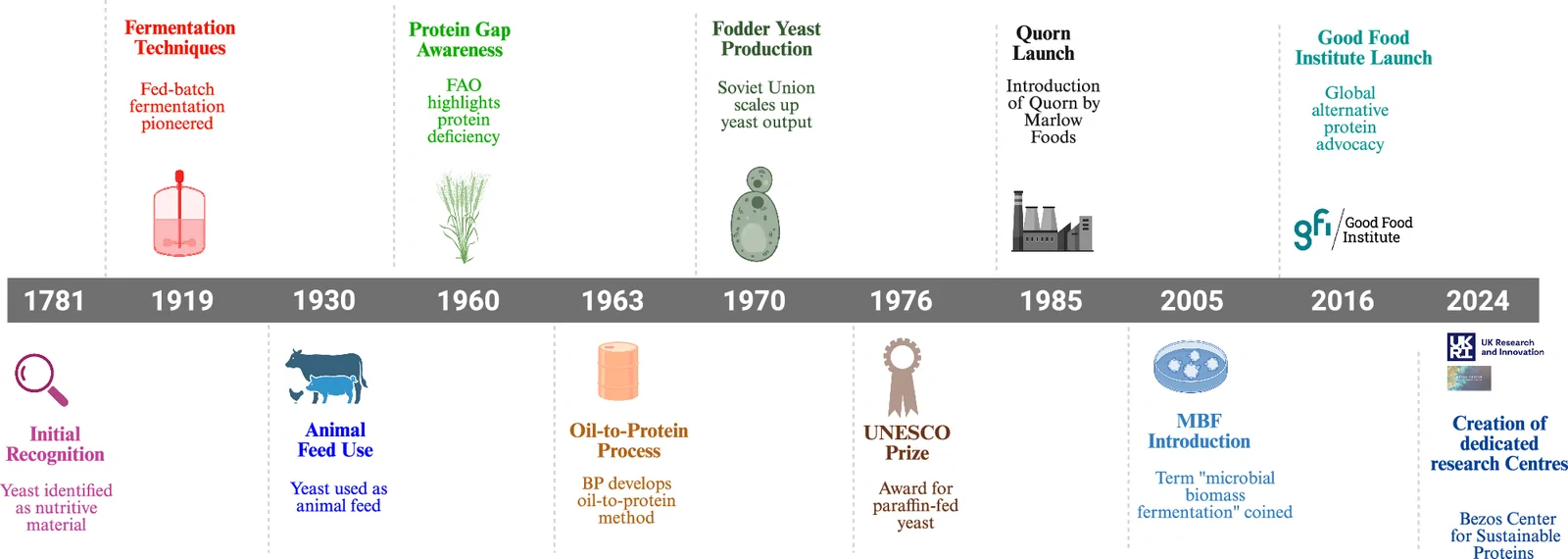
Historical milestones towards modern microbial biomass fermentation from 1781 to 2024. The timeline highlights the recognition of yeast as a nutritive material in 1781; the pioneering of fed-batch fermentation in 1919; the development of animal feed and oil-to-protein applications; the launch of UNESCO and Quorn; the coining of the term “microbial biomass fermentation” in 2005; and recent advances in sustainable production.

**Fig. 2:**
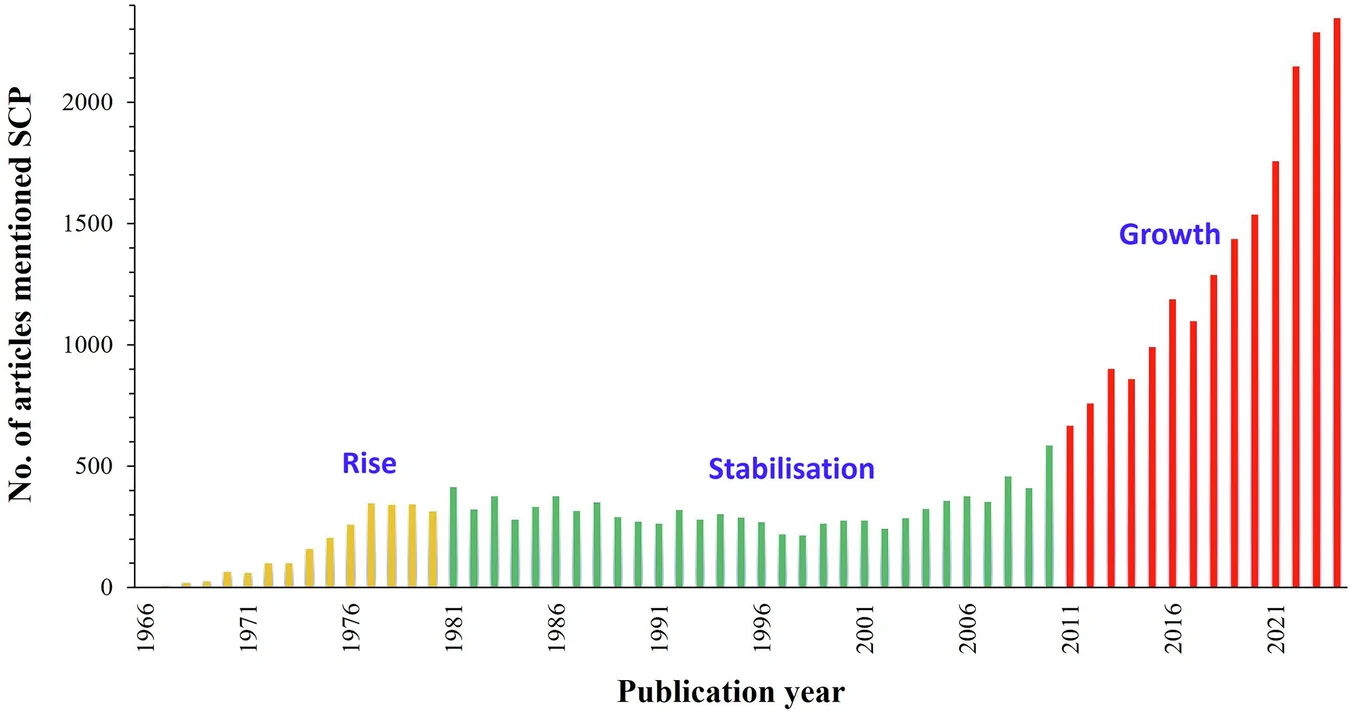
Evolution of single-cell protein (SCP) research from 1966 to 2024 based on Google Scholar analysis. The figure shows the number of articles mentioning SCP from 1966 to 2024, illustrating a rise in interest during the 1970s, a stabilisation period through the 1980s-1990s, and exponential growth in publications since 2010, peaking at 2,300 articles by 2024, highlighting the phases ‘Rise of SCP’, ‘Stabilisation’, and ‘Exponential growth’.

Box 1 Microbial proteins: History Humanity has harnessed microbial fermentation since at least 7000 BC for bread and beverages across Egypt, China, Greece, and the Roman Empire. The era of single-cell protein (SCP) began in 1781 (Fig. 1), when baker’s yeast (*Saccharomyces cerevisiae*) was first recognised as a concentrated source of nutrients. In 1919, Sak and Hayduck established fed‑batch fermentation, and by the 1930s, Max Delbrück demonstrated the feed value of surplus brewer’s yeast. Post‑war concerns about malnutrition and FAO’s 1960 “protein gap” report catalysed global investment in microbial proteins. In the 1960s-1970s, British Petroleum’s “proteins‑from‑oil” process, Soviet hydrocarbon yeasts, and UNESCO’s 1976 prize exemplified industrial ambition^61,69^. Many ventures collapsed by the 1980s due to high costs, complex downstream processing, consumer hesitancy, and the easing of food shortages caused by the Green Revolution (Fig. 2)^34^. Quorn™ (microbial product derived from the fungus *Fusarium venenatum*), launched in 1985, demonstrated the competitiveness of microbial proteins and remains one of the few long‑standing commercial successes^32,33^.

**Fig. 3:**
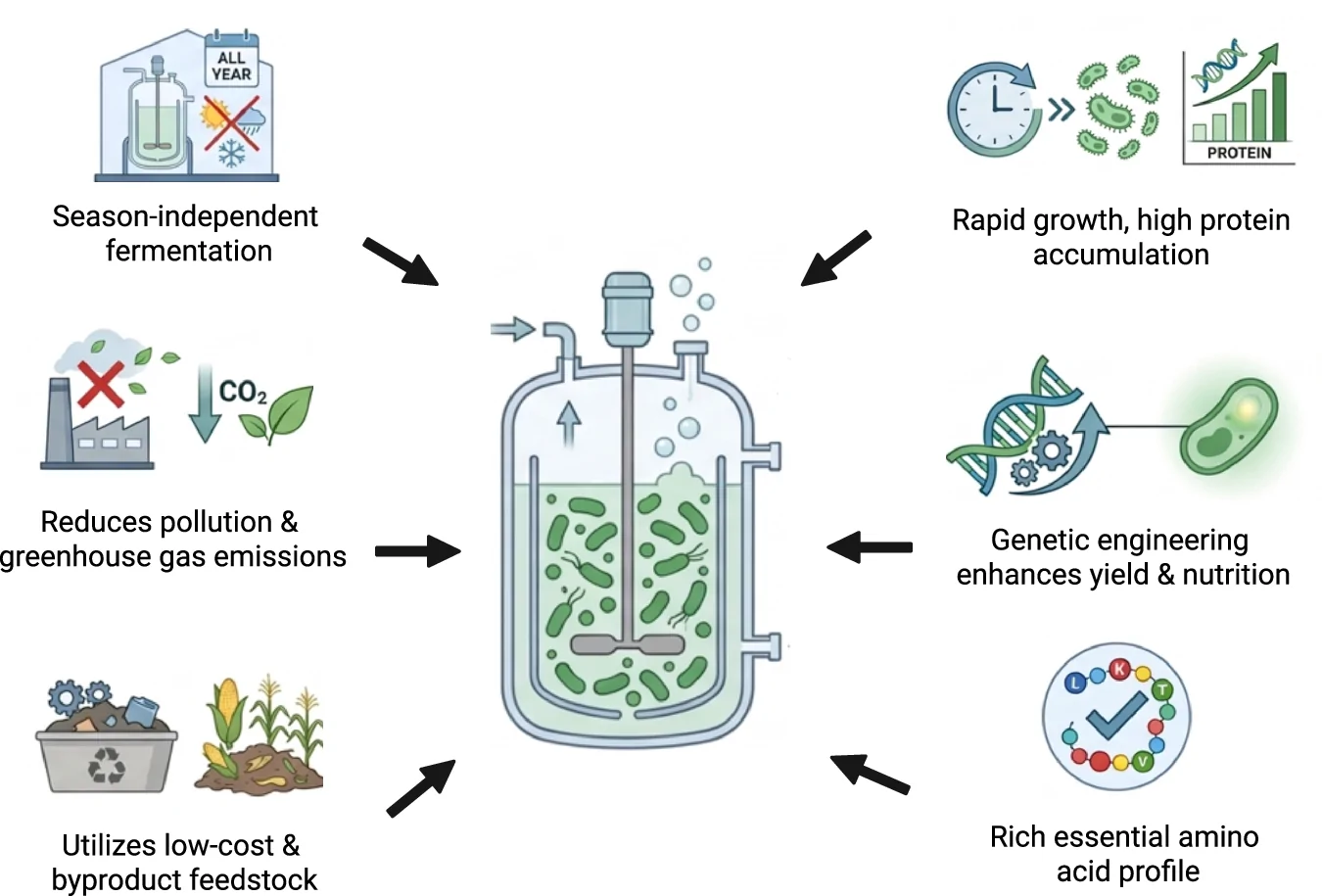
Advantages of microbial biomass fermentation. The figure highlights 6 core benefits. Rapid microbial growth enables high protein accumulation, whereas targeted genetic engineering further boosts yield and nutritional value. The resulting biomass offers a rich profile of essential amino acids and can be produced continuously using low-cost, locally sourced waste feedstocks. This process can reduce pollution and GHG emissions, use relatively little land and water, and offer a scalable platform for future protein production, particularly when coupled with low-impact feedstocks such as C1 substrates, residues, or other non-food inputs.

Box 2 Microbial proteins: Opportunities Microbial proteins are commonly produced via microbial biomass fermentation, which offers several advantages over conventional plant and animal proteins (Fig. 3)^40^. Representative microorganisms used for microbial protein production include yeasts such as *Saccharomyces cerevisiae*, *Candida utilis*, and *Yarrowia lipolytica*; filamentous fungi such as *Fusarium venenatum*; bacteria including hydrogen-oxidising and methanotrophic species; and microalgae such as *Arthrospira* (spirulina) and *Chlorella*^12,13,47^. Microbial cultures grow exceptionally fast, with doubling times on the order of hours, accumulate high levels of cellular protein (30-70% w/w), and can be tailored through genetic modification, evolution, and selection to enhance yield and nutritional quality^47,60^. Microbial biomass fermentation can valorise a wide range of low-cost, locally abundant biological feedstocks and side streams, as well as CO₂ and other C1 substrates (single-carbon feedstocks such as CO₂, methane, methanol, and formate). In doing so, it reduces environmental pollution and GHG emissions while lowering input costs^38,70^. Importantly, microbial biomass fermentation has the potential to be integrated with existing farming systems by valorising residues such as crop straws, corn stover, and sugarcane bagasse into higher-value protein products. However, realising this potential requires dedicated pretreatment and fermentation infrastructure, robust supply logistics, and food-grade process standards^40,63^. In doing so, it upgrades low‑value by‑products into additional income sources for farmers while reducing residue disposal and agricultural waste^46^. Continuous and non-continuous fermentation processes operate independently of seasonal or climatic fluctuations. Compared with ruminant meat, they can reduce land and water use by around 90% per kilogram of protein. Scenario modelling further shows that substituting just 20-80% of global ruminant meat demand with microbial proteins by 2050 could cut annual deforestation by 56-93% and net CO₂ emissions from land-use change by 56-87%^28,37,39^. Moreover, they generate far less residual waste (60-90%) than traditional food‑production systems^1^. Microbial biomass fermentation is highly resilient, ensuring a stable protein source even during extreme food supply crises^43,62^. Historically, microbial proteins have been mobilised during wartime to mitigate protein shortages, for example, *Candida utilis* yeast grown from paper waste during World War II.

**Fig. 4:**
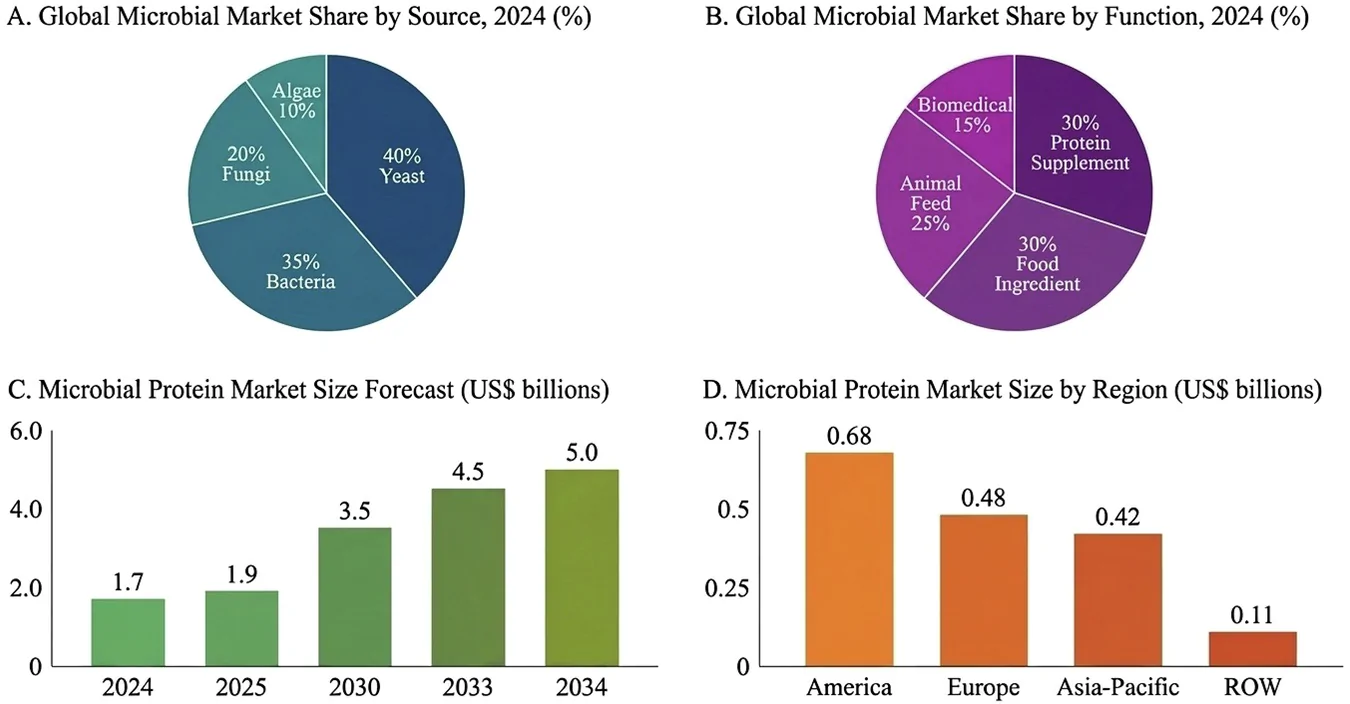
Global microbial protein market (food and animal feed) overview. **A** Market share by microbial source, **B** Functional distribution of microbial protein. **C** Projected market size from 2024 to 2034. **D** Regional breakdown for 2024. Data source: Market Research Future - MRFR, Global Microbial Protein Market, Market Analysis 2019-2034. ROW: rest of the world.

Box 3 Microbial proteins: Market dynamics Commercial alternative proteins are still dominated by plant-based products (88.9% in 2023), which account for the vast majority of global sales, whereas microbial proteins (8.2%), insect-based proteins (2.7%), and cultivated meat and seafood (0.2%) segments remain comparatively small and nascent^44^. The market is expected to expand from USD 15.3 billion to USD 26.5 billion between 2023 and 2030, with a compound annual growth rate (CAGR) of 8.2%^44,71^. Microbial proteins were valued at ~USD  1.7 billion in 2024 and are expected to reach USD ~ 1.9 billion in 2025 and USD ~ 5.0 billion by 2034, with a CAGR of ~11% (Fig. 4).

### A three-phase adoption model

Consumer acceptance of microbial protein is shaped by a constellation of psychological, demographic, cultural, and informational factors rather than by a single attribute^7^. At the individual level, acceptance hinges on perception, flavour expectations and experiences, trust in producers and regulators, emotional responses, attitudes, and purchase intention^19–21^. Demographics further modulate these effects, including geographic region, gender, age, education level, and existing dietary practices^22,23^. Adoption is also influenced by barriers such as price sensitivity, food neophobia and concerns about displacing traditional agriculture^24–26^.

**Fig. 5:**
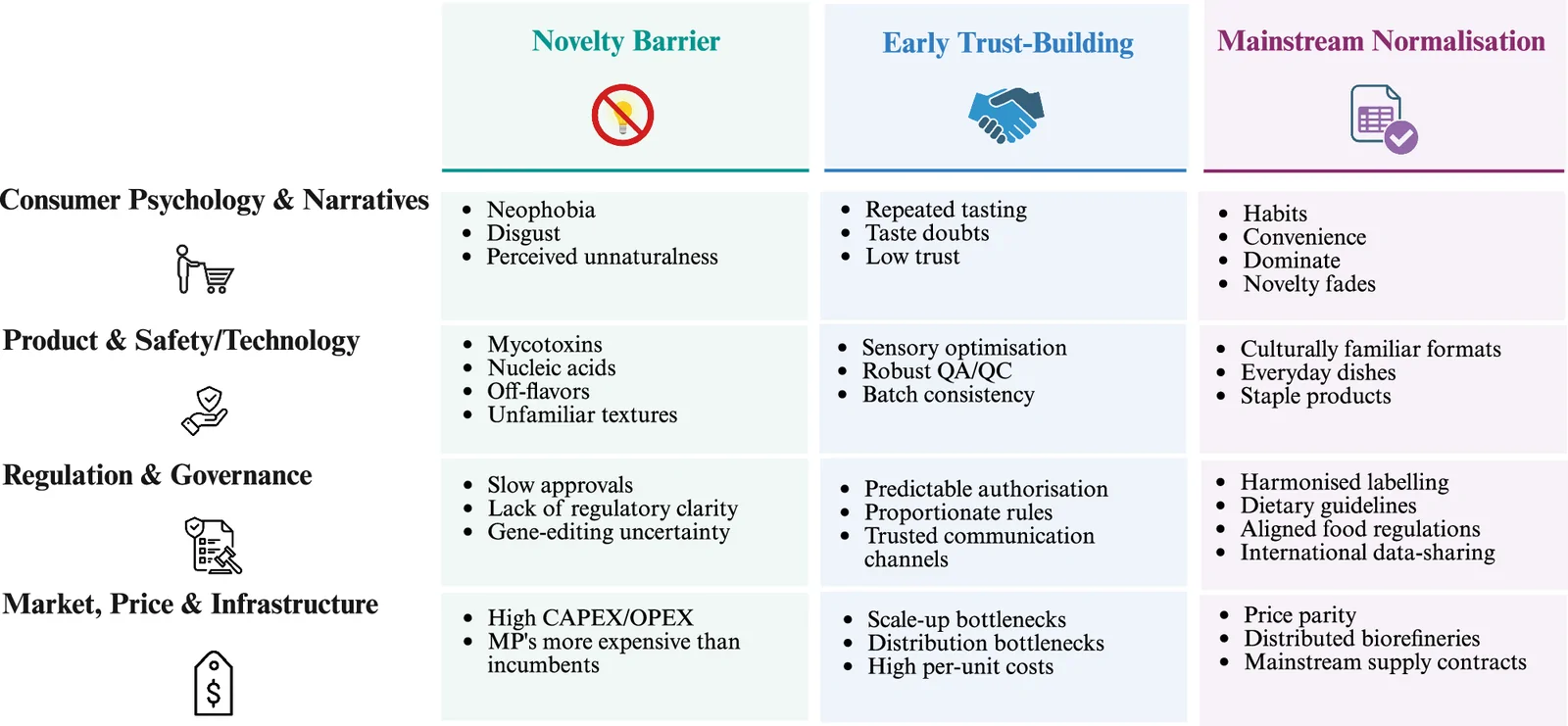
Actionable adoption framework for microbial proteins. A three-phase trajectory (novelty barrier, early trust-building, mainstream normalisation) is linked to four domains of intervention: consumer psychology and narratives; product and safety/technology; regulation and governance; and market, price, and infrastructure. Each cell highlights dominant barriers and illustrative levers for change. CAPEX/OPEX: Capital Expenditure/Operating Expenditure.

We propose a three‑phase adoption process: the novelty barrier, early trust-building and mainstream normalisation. These phases and associated levers are summarised in Fig. 5. While regulatory approval remains a necessary prerequisite for market entry, evidence from food-grade microbial protein case studies suggests that consumer acceptance often becomes the dominant constraint once safety and compliance have been established. The novelty barrier is dominated by neophobia and perceptions of unnaturalness and risk, often manifesting as disgust. A survey conducted in the UK, Germany and Romania found that 47.1% of respondents exhibited neophobia toward fungal protein, often citing associations with spoilage and excessive processing. Respondents who reported disgust reactions were significantly less willing to purchase these products^27^. This indicates that regulatory approval alone is insufficient to drive adoption and that familiarity must instead be engineered through early exposure and sensory masking. Microbial proteins should not be framed as a uniform category; rather, individual products should be assessed based on their specific nutritional, safety, sensory, and environmental characteristics^27^.

In the early trust‑building phase, transparency about production processes, consistently positive sensory experiences, and alignment with shared challenges such as climate change^28^, food security^29^, and dietary health^30^ are critical. Trusted intermediaries, such as public health authorities, can legitimise the technology. However, sensory experience remains a gatekeeper. Consumers with strong preferences for familiar sensory attributes are 46-56% more likely to choose animal meat and 25-26% less likely to choose cell-cultured meat. Plant-based options may face less resistance than newer alternative protein categories, partly because they benefit from a longer history of consumption, greater product familiarity, and stronger integration into existing culinary practices^25,31^.

Mainstream normalisation occurs when microbial proteins achieve price parity, cultural integration, and sustained retail presence. At this stage, novelty fades, and products are judged by conventional metrics of value, flavour, accessibility, and ethical alignment within existing food‑system infrastructures. Quorn™ mycoprotein, launched in the UK in 1985 and now sold in 20 countries with billions of servings consumed, exemplifies how a fungal mycoprotein can become an established, everyday meat alternative^32,33^. Other microbial-derived products demonstrate parallel routes to market acceptance, including spirulina, an established biomass-based food ingredient, and SCOs, an emerging category of microbial fats with potential applications as sustainable alternatives to conventional oils and speciality fats^12,13,18^. Ultimately, advancing microbial protein requires an interdisciplinary approach. This should couple microbial engineering with food sociology, behavioural science, and strategic communication design. Such integration can better align scientific innovation with evolving psychological and cultural expectations. In the following sections, we connect these phases to specific technological, regulatory, and policy barriers and enablers.

### Technological and economic challenges

Early microbial protein ventures, such as the Imperial Chemical Industries (ICI) programme, succumbed to competition from cheaper agricultural proteins and escalating maintenance expenses^34,35^. High capital expenditure for advanced bioreactors, downstream processing equipment, and facility infrastructure continues to place microbial proteins at a cost disadvantage relative to conventional agricultural proteins^36^. These capital barriers primarily impede the shift from early trust-building to mainstream normalisation. They delay the point at which microbial proteins can compete with established animal and plant proteins on shelf price, product variety, and distribution.

Operating expenses, driven by feedstock pretreatment (especially lignocellulosic residues), energy‑intensive sterilisation, aeration, and purification, inflate unit costs and challenge profitability^37^. Feedstock choice is also critical from a systems perspective: microbial-protein production should ideally avoid direct competition with current food and feed supply chains. In this regard, non-food carbon sources, particularly C1 substrates such as CO₂, methane, and methanol, are especially promising because they can decouple biomass production from arable land, sugar crops, and conventional feed inputs, although their use remains organism- and process-dependent^1,28,38,39^.

In low- and middle-income settings, complex pretreatment and energy-intensive unit operations can constrain the inclusion of microbial proteins in public procurement schemes for schools, hospitals, or social protection programmes. However, institutional procurement is only one possible route to scale. Other pathways may include use as functional food ingredients, incorporation into blended products, supply through food-service and retail channels, or deployment through regional processing hubs tailored to local feedstocks and markets. These constraints nonetheless slow broader commercial expansion and the transition from pilot-scale production to widespread use^40^.

Furthermore, ensuring consistent product quality demands tight control of fermentation parameters and stable strains. For example, food-grade fungal biomass must be produced under tightly controlled pH, temperature, aeration, and substrate conditions to maintain reproducible protein composition and avoid contaminant formation, while additional downstream processing may be required to reduce nucleic acid content. Established mycoprotein producers such as Quorn™ use a heat-treatment step to activate endogenous RNases and reduce RNA to below 2%, illustrating how quality and safety specifications can impose additional process constraints during development^32^. These criteria can prolong development timelines and require a large investment in research and development (R&D)^41^. Prolonged timelines and high R&D costs reinforce perceptions that microbial proteins are experimental and high-risk. This dampens investor appetite and reduces retailer willingness to allocate scarce shelf space during the novelty and early trust-building phases^42^. This risk is reflected in the experience of several recent ventures in the sector, where promising technical platforms have nonetheless struggled to sustain commercial momentum due to the combined pressures of high R&D intensity, complex scale-up, and constrained access to capital, as seen in the case of the Swedish mycoprotein company Mycorena^43,44^.

High upfront capital costs remain a major constraint, particularly for first-of-a-kind production facilities that must demonstrate both technical performance and regulatory readiness before reaching commercial scale. Investor willingness to provide such capital depends not only on projected production costs, but also on the degree to which scale-up, regulatory, and market risks can be reduced. Shared pilot and demonstration facilities, public co-funding, clearer regulatory pathways, strategic procurement commitments, and the possibility of repurposing existing fermentation infrastructure can all improve the investment case by lowering perceived risk and shortening the path to revenue^28,34,43,44^.

For many companies, the challenge is not only high CAPEX in principle but also access to CAPEX in practice. Financing risk favours platforms that can retrofit or repurpose existing fermentation infrastructure rather than those that require greenfield facilities.

Intellectual property (IP) also shapes commercial trajectories in this sector. On one hand, patents, proprietary strains, and protected process know-how can help firms attract investment and secure routes to market. On the other hand, fragmented IP landscapes, licensing costs, and restricted access to enabling technologies may increase development costs and slow follow-on innovation. These dynamics can be especially challenging for early-stage companies and smaller manufacturers, which may lack the capital needed to build greenfield facilities or secure broad access to technology. In practice, this may favour platforms that can operate within adapted existing fermentation infrastructure, rely on shared pilot and contract-manufacturing capacity, or integrate into regional production hubs^34,44–46^.

Multiple converging trends offer a more optimistic outlook. Anticipated disruptions in traditional agriculture due to climate change, land degradation, and resource limitations are likely to shift the cost balance in favour of microbial proteins. Simultaneously, as additional fermentation facilities become available, economies of scale will reduce operational costs. Advances in feedstock optimisation, process intensification, and AI-guided bioprocess control are also expected to reduce energy inputs and improve yield predictability. Continued progress in strain engineering and fermentation analytics may shorten development cycles, enhance product stability, and ultimately improve economic viability^45,47^. These advances can bring microbial proteins closer to price parity and reduce production volatility, thereby supporting the transition from early trust-building to mainstream normalisation.

### Safety and regulatory considerations

Safety and regulatory considerations present significant structural barriers. The potential for mycotoxin, pathogen, or heavy-metal contamination in animal and human feeds, particularly given the capacity of some microbial strains to biosynthesise toxic secondary metabolites, necessitates rigorous analytical quantification and exhaustive downstream screening. Depending on the organism, substrate, and production process, additional undesirable compounds, including antinutritional factors (e.g., saponins or cyanogenic glycosides), may also need to be monitored and controlled. It also requires comprehensive in vivo validation with animal and human^39,48^. In human foods, excessive nucleic acids can elevate blood uric acid levels, predisposing consumers to hyperuricaemia, gout, nephrolithiasis and cardiovascular complications^49^. Residual live cells pose a risk of opportunistic infections, and inadvertent toxin synthesis requires meticulous strain selection^30,41^.

It is important to note that microbial proteins span a heterogeneous group of products. Biomass derived from well-characterised food organisms such as *Saccharomyces cerevisiae* or other taxa with established food use differs materially, from both regulatory and consumer-acceptance perspectives, from products based on less familiar organisms such as certain marine bacteria, methanotrophs, or newly isolated fungi^12,42^.

Microbial protein producers must meet rigorous safety standards that cover identity, compositional analysis, toxicology, allergenicity and intake estimates^49,50^. Many of these safety and quality-control challenges are not unique to microbial fermentation. Comparable issues are routinely managed in traditional fermented foods and beverages, as well as in broader food production systems, where microbial contamination, toxin formation, and process hygiene must also be controlled. Familiar examples include fermented dairy products, soy fermentations, and alcoholic beverages^51,52^. The distinction lies in the regulatory unfamiliarity of microbial systems rather than their intrinsic risk. The establishment and harmonisation of clear, science‑based regulations will streamline the market introduction of microbial proteins^47^.

In the EU, a critical regulatory distinction is whether a food was consumed to a significant degree before 15 May 1997^53^. Products without such a history generally fall under the Novel Food framework and require a substantially more demanding authorisation process, including a well-defined production process, compositional characterisation, exposure assessment, and, where necessary, toxicological evidence. In practice, this can require companies to produce regulatory-grade material while the process is still being optimised, lock down a specific production strain and process configuration for dossier consistency, and absorb multi-year approval timelines before revenue generation begins. These requirements can create a marked disadvantage for genuinely novel microbial protein platforms relative to established food organisms and ingredients. In the United States, market entry may proceed through GRAS pathways for some ingredients, but this depends strongly on the organism, intended use, and available safety evidence, meaning regulatory burden still varies substantially across microbial protein categories^50^.

### Emerging technical and infrastructural solutions

Emerging technical innovations now enable targeted amplification of protein biosynthetic pathways, including through CRISPR-Cas9 genome editing. At the same time, non-GMM approaches such as adaptive laboratory evolution (ALE), strain isolation, and classical selection also provide important routes to improving substrate utilisation, robustness, productivity, and nutritional performance^45,47^.

To control potential risks associated with genetic manipulation, tailored regulatory frameworks must be established^47^. These frameworks must differentiate between traditional genetically modified microorganisms (GMMs) and newer gene-edited strains. The latter are developed through precise genetic techniques, such as CRISPR-based modifications that do not involve the insertion of foreign DNA. In some jurisdictions, regulatory treatment has begun to differentiate certain gene-edited products from conventional genetically modified organisms. For example, in the UK, some precision-bred organisms may fall outside the stricter GMO framework when the changes could have arisen through traditional breeding, while in Japan, genome-edited foods without inserted foreign DNA are subject to a notification-based approach rather than the same pathway used for transgenic GM foods. In the United States, foods from genome-edited plants are regulated under existing food-safety authorities using a risk-based, product-specific approach^54–57^. This reflects a shift towards product-based rather than process-based assessments^50^. This distinction has implications for strain approval processes, labelling requirements and public acceptance. Faster approval and transparent labelling can also accelerate early trust-building and compress timelines to mainstream normalisation for products derived from these microbes.

Process intensification through continuous fermentation and modular bioreactors can reduce operating costs and facilitate scale‑up^36,43^. Continuous fermentation can improve volumetric productivity in suitable systems, but it also increases operational demands for sterility assurance, process monitoring, cleaning strategies, and upstream/downstream integration. It is therefore not a universal first-choice for scale-up and may be preferable only when the biological system and process economics clearly justify the added complexity^18,43^. The use of multistage continuous bioreactors and airlift systems to achieve high volumetric productivities for amino acids and enzymes while maintaining food-grade standards shows the realisation of these strategies at scale. To address excessive nucleic acids, established mycoprotein producers such as Quorn™ apply a heat treatment to activate endogenous RNases, which degrade cellular RNA^32^. Approaches include metabolic incorporation of desirable flavour compounds, iterative formulation refinement with food scientists and chefs, and strategic co-culture or blending of complementary strains^58^.

Regulatory pathways can be streamlined by utilising generally recognised as safe (GRAS) microorganisms and applying risk-based downstream processing strategies that meet food-grade purity thresholds, including the removal or control of living cells, foreign DNA and RNA, residual media components, solvents, and other process-related impurities, without requiring the stringent purification standards typical of pharmaceutical products. A further practical constraint is that applicants typically need to anchor the dossier to a defined production strain and process specification. This can effectively pause iterative strain improvement during regulatory review, creating a tension between ongoing optimisation and dossier consistency. Maintaining transparent safety standards that document strain lineage, processing methods, and toxicological assessments is essential^50^.

Public-facing education campaigns should emphasise not only health benefits, sustainability credentials, and clear ingredient and nutritional labelling, but also how consumers can prepare and incorporate these products into familiar meals. In this context, food scientists, product developers, and chefs can play a key role in improving formulations, communicating use cases, and building culinary familiarity^59^. For example, long-running communication and labelling strategies around commercial mycoprotein products have normalised fungal biomass as a food ingredient in several markets, contributing to sustained consumer uptake despite initial perceptions of novelty^26,59^. Related precedents already exist within the food system. Yeast-derived products, including yeast extracts and nutritional yeast, are widely used and familiar to consumers, while spirulina provides an example of microbial biomass that has achieved market presence as both a supplement and food ingredient. These examples illustrate that microbial-derived foods can, under the right regulatory and market conditions, become normalised^12,58,60^.

Many current fermentation systems remain energy-intensive because they rely on sterilisation, agitation, aeration, cooling, and downstream processing, and are often coupled primarily to grid electricity rather than dedicated low-carbon energy sources. The relative importance of these factors depends on the organism, the process design, the site configuration, and the product specification^18,43,48^. Emerging efforts to integrate fermentation with renewable energy platforms, such as solar, wind, or anaerobic digestion, could reduce carbon footprints and buffer producers against volatile energy costs. Similarly, while most microbial protein production remains centralised, decentralised biorefineries located close to biomass sources have the potential to minimise transport costs, expand rural access to bio-derived foods, and strengthen local circular economies^46,61^. Embedding carbon‑capture technologies within these workflows further maximises resource efficiency, valorises by‑product streams and advances multiple UN Sustainable Development Goals, notably SDG 2 (Zero Hunger), SDG 9 (Industry, Innovation and Infrastructure), SDG 12 (Responsible Consumption and Production), and SDG 13 (Climate Action)^61,62^.

In parallel, feedstocks can be diversified beyond conventional sugars by harnessing C1 substrates (e.g., CO₂, methane, methanol), lignocellulosic residues, and food waste, thereby reducing direct competition with food and feed production chains. Lignocellulosic residues are attractive in principle but often require costly and technically demanding pretreatment before efficient microbial conversion. Food-waste streams can reduce raw-material costs, yet they introduce challenges related to compositional variability, contamination risk, and regulatory acceptability for food applications. By contrast, C1 substrates can avoid direct competition with food crops, but only a limited subset of organisms can utilise them efficiently^39^. However, access to C1 substrates at scale requires enabling infrastructure, including CO₂ capture or concentration, gas purification and compression, safe storage and distribution, suitable gas-handling bioreactors, and a reliable, low-carbon electricity supply. The relevance of these pathways to lower-resource settings is therefore context dependent: they may be most viable where biogas, industrial off-gases, wastewater-treatment streams, or concentrated CO₂ sources are already available, or where regional energy and industrial systems can support gas-based biomanufacturing^38,48^. This diversification can expand resource availability, reduce raw material costs, and enhance the overall sustainability profile of microbial protein production^38,63^.

We map these enablers onto our three-phase framework in Fig. 5. Within this framework, these technical and infrastructural recommendations help lower apparent risk and improve performance during the novelty and trust-building phases. They also drive the cost and availability gains required for durable mainstream normalisation.

### Policy recommendations

Policy will play a central role in determining whether microbial proteins become marginal novelties or embedded pillars of future food systems. We advocate a three‑theme agenda focused on infrastructure and skills, regulatory modernisation, and demand stimulation^64^.

First, sustained public and private investment in medium‑ to large‑scale fermentation infrastructure hubs, such as the Bezos Centre for Sustainable Protein or the Microbial Food Hub, can bridge the gap between laboratory innovation and commercial deployment. Major international initiatives illustrate this model, including the Centre for Process Innovation (CPI) in the UK, BioMADE in the United States, the FoodPilot network in the EU, and Singapore’s Food Innovate facilities. Similar functions can also be delivered in emerging economies through regional shared facilities, university-industry consortia, contract manufacturing, retrofitted existing fermentation assets, and modular production systems. In these contexts, the goal is not necessarily to replicate large capital-intensive models, but to create locally appropriate pathways that reduce scale-up risk, support food-grade process development, and connect microbial-protein innovation to available feedstocks, skills, and markets^43,46,64^. These shared pilot- and demonstration-scale facilities will reduce scale-up risk, shorten learning curves, and lower capital barriers. By providing access to equipment, expertise, and regulatory guidance for startups, researchers, and established firms, these hubs create robust support structures. They also catalyse the formation of regional clusters of biomanufacturing excellence. Such hubs may also play an important role in integrating smaller manufacturers into the ecosystem. Rather than assuming that microbial-protein production will be dominated solely by large firms, shared infrastructure, toll manufacturing, and modular processing could enable small- and medium-sized enterprises and regional producers to participate in ingredient production, formulation, or context-specific deployment, particularly where local feedstocks or specialised markets are relevant^40,43,46^.

Second, regulatory modernisation should aim for clarity, proportionality, and adaptability. Well‑resourced regulators require stable funding, scientific training, and mechanisms for horizon scanning to keep pace with fast‑moving microbial technologies. Streamlined and harmonised procedures for assessing microbial biomass fermentation will reduce uncertainty without compromising safety. For example, Fermotein, a mycoprotein derived from *Rhizomucor pusillus* biomass, has recently received a positive scientific opinion from the European Food Safety Authority under the EU Novel Foods Regulation, demonstrating progress in navigating existing frameworks for whole-biomass microbial food ingredients^53^ rather than relying solely on genetically engineered strains. Aligning food and feed regulations and, where appropriate, recognising histories of safe use would facilitate cross-border trade and investment. Promoting international data-sharing would further support this process^47,50^.

Third, demand‑side policies can help microbial proteins move from the niche to the mainstream. Public procurement for schools, hospitals, prisons, and the military can create stable markets for microbial‑enriched products that meet nutritional and sustainability criteria. Fiscal incentives, such as reduced value-added tax (VAT), a consumption tax applied to most food products in many jurisdictions, or targeted subsidies for lower-impact proteins, can directly lower retail prices, improving price competitiveness relative to conventional animal-derived proteins and other alternative protein products. At the same time, communication campaigns are critical. They should couple affordability with clear messaging on health, climate, and animal-welfare benefits, while also positioning products in familiar culinary formats and flavour frames, since novel flavour profiles are often better accepted when linked to recognisable market references and usage occasions^19,59,65–67^. In these contexts, adoption barriers centre on affordability, reliability, and cultural fit under conditions of food insecurity, rather than on lifestyle environmental concerns or protein oversupply. When coupled with decentralised biorefineries that valorise locally available residues and C1 streams, procurement schemes can support rural livelihoods and increase access to nutrient-dense foods. Explicitly targeting affordability and equitable access is essential to prevent microbial proteins from remaining niche products for affluent urban consumers. However, this also requires capacity building, enabling infrastructure, and process adaptation to local conditions, so that production and distribution systems can align with available feedstocks, technical capabilities, energy systems, and market needs. Together, these levers can lock in microbial proteins as enduring components of sustainable food systems rather than transient innovations.

### Future perspectives

Microbial biomass fermentation can contribute to more sustainable and resilient food systems, but its long-term role will depend on more than technical feasibility^68^. Future work should determine where microbial proteins provide the greatest nutritional, environmental, economic, and social value, and under which production and policy conditions these benefits can be realised. This requires integrated assessment of feedstocks, energy sources, process design, safety, sensory quality, affordability, and cultural fit.

A key priority is to generate stronger empirical evidence on adoption over time. Longitudinal consumer studies are needed to test how familiarity, repeated exposure, transparent labelling, culinary integration, and trusted communication shape acceptance beyond initial novelty. Policy and procurement experiments will also be important for evaluating whether schools, hospitals, public institutions, and regional food programmes can support stable demand while improving affordability and access.

Several outstanding technical and regulatory questions remain. More evidence is needed on scalable, low-impact feedstocks, especially C1 substrates, residues, and food waste streams, as well as on the infrastructure required to use them safely and economically. Regulatory systems will need to balance rigorous safety assessment with proportionate, predictable pathways that do not unnecessarily delay responsible innovation. Greater clarity around strain fixation, dossier requirements, histories of safe use, and international data-sharing could reduce uncertainty for developers and investors.

Future development should also avoid assuming a single global pathway to scale. In some settings, large centralised facilities may be most efficient, whereas in others, retrofitted fermentation assets, regional hubs, contract manufacturing, or modular systems may better align with local feedstocks, skills, energy systems, and markets. Understanding these differences will be essential if microbial proteins are to support food security and reduce protein-price volatility in climate-vulnerable, import-dependent, or resource-constrained regions.

The three-phase framework proposed here, novelty barrier, early trust-building, and mainstream normalisation, offers a basis for testing which interventions matter most at different stages of adoption. Future research should refine this framework using comparative case studies, techno-economic analyses, regulatory evidence, and real-world market data. Such work will help determine whether microbial proteins remain niche products or become durable components of future food systems.
